# 一项针对原发免疫性血小板减少症的医患调研：I-WISh国际调研中国亚组分析

**DOI:** 10.3760/cma.j.issn.0253-2727.2021.05.004

**Published:** 2021-05

**Authors:** 茹婷 王, 新光 刘, 宇 侯, 明 侯

**Affiliations:** 山东大学齐鲁医院血液科，济南 250012 Department of Hematology, Qilu Hospital, Shandong University, Jinan 250012, China

**Keywords:** 免疫性血小板减少症, 生活质量, 情绪影响, 治疗认知, Primary immune thrombocytopenia, Quality of life, Emotional impact, Treatment cognition

## Abstract

**目的:**

探讨原发免疫性血小板减少症（ITP）对患者生活质量和情绪健康的影响及医患双方对治疗方法的认知。

**方法:**

该项研究为I-WISh国际调研的中国亚组分析，共102名医生和286例ITP患者参与调研，调研结果采用描述性分析。

**结果:**

疲劳和对血小板计数不稳定的焦虑是ITP患者在治疗后的主要症状，而医生对患者疲劳、焦虑症状的报告不充足。超过90.0％的患者认为ITP对他们的工作或学习、日常事务、体能、运动能力和性生活产生了负面影响。60.8％（174/286）的患者认为ITP对情绪影响较大，其中对血小板计数波动（74.8％，214/286）、疾病本身（71.7％，205/286）及病情恶化（68.9％，197/268）的担忧占主要方面，医生也给出了类似的评价。医患双方均认为减少自发出血、维持安全稳定的血小板水平和改善生活质量是ITP最重要的三个治疗目标，其中降低出血风险是影响医生治疗决策的首要因素。此外，医生认为糖皮质激素（54.9％，56/102）最有可能使患者达到持续有效的治疗目标，而患者对血小板生成素受体激动剂（TPO-RA）的反馈最好，且83.9％（240/286）的患者对TPO-RA的疗效表示满意。

**结论:**

ITP对患者生活质量和情绪存在巨大影响，但中国医生对ITP患者疲劳和焦虑症状认识不足。在ITP诊治过程中医患应加强沟通，共同决策，以制定合适的个体化治疗方案。

## Abstract (en)

**Objective:**

To explore the impact of primary immune thrombocytopenia（ITP）on patient's quality of life and emotional well-being, and both the physician and the patient's perception of the treatment.

**Methods:**

This study was a Chinese Subgroup analysis of I-WISh survey, including 102 physicians and 286 ITP patients invited to complete the survey. A descriptive analysis of the results of the physician-patient survey was conducted in three parts: symptoms, impact of ITP on quality of life and emotion, and treatment perception.

**Results:**

Fatigue and anxiety about unstable platelet counts were the main symptoms after treatment. Physicians reported inadequate frequency of anxiety and fatigue. In terms of quality of life, over 90.0％ of patients thought that ITP had a negative impact on their working life/studies, daily tasks, energy, capacity to exercise, and sexual life. With regard to emotion, 60.8％（174/286）patients thought that ITP had a greater impact on overall emotion，concerns about the fluctuation in platelet level（74.8％, 214/286）, disease itself（71.7％, 205/286）and disease progression（68.9％, 197/286）were the common issues, which were similar to the physicians' evaluations. Both physicians and patients agreed that reducing spontaneous bleeding, maintaining a healthy blood count and improving quality of life were the top three important treatment goals. Physicians considered reducing bleeding risk as the most important factor for decision making. Physicians believed that patients receiving glucocorticoid（54.9％, 56/102）were most likely to succeed in achieving sustained response while patients had the best response to thrombopoietin receptor agonist（TPO-RA）treatment, with about 83.9％（240/286）being satisfied with the overall effectiveness of TPO-RA.

**Conclusion:**

Physicians in the I-WISh Chinese subgroup had low awareness on patients' fatigue and anxiety. The quality of life and emotion of patients were significantly negatively affected by ITP. There are differences in the selection of treatments between physicians and patients, suggesting that physicians should raise concerns about the quality of life of ITP patients and make individualized treatment options through physician-patient shared decisions.

原发免疫性血小板减少症（primary immune thrombocytopenia, ITP）是一种以血小板减少为特征的自身免疫性疾病，是临床最常见的出血性疾病。ITP患者中轻者可表现为无症状血小板减少、皮肤黏膜出血，重者可出现消化道甚至颅内出血，威胁患者生命健康。乏力是ITP患者最常见的非出血性临床表现[1]。

ITP对患者的生活质量有着多方面的影响，广泛涵盖情绪健康、工作生活及其他日常活动_[2]_，并可能消耗大量医疗资源（如为了解决急性出血事件而频繁住院），因此ITP患者健康相关生活质量常明显下降[3]。近年来随着“以患者为中心”医疗模式的不断推进，健康相关生活质量已成为衡量ITP患者治疗效果的重要指标之一[4]。因此做好症状管理、提升患者生活质量对于ITP患者尤为重要。加强医生和患者对ITP治疗的认知有助于制定个体化治疗方案，进一步改善患者预后。本研究报道了一项针对中国ITP患者及血液科医生的调研结果，以期为改善中国ITP治疗现状提供帮助。

## 对象与方法

一、研究对象

ITP全球影响调研（I-WISh）是一项针对来自13个国家的血液科医生和ITP患者的临床调研，于2017年12月至2018年5月进行。医生由当地田野调查机构邀请，要求能自主决定ITP患者治疗方案且已诊治3例以上的ITP患者。患者由经治医生或患者支持组织邀请，需≥18岁且确诊为ITP。本研究为I-WISH中国亚组分析，共有102名医生和286例ITP患者（170例由患者支持组织邀请，116例由经治医生邀请）参与调研。

二、调研

纳入医生和患者均完成约30 min的在线或纸质调研，调研问卷由多名医生和ITP患者权益倡导者组成的指导委员会设计，其内容包括人口学资料、症状、诊断、ITP对生活质量和情绪的影响、ITP对工作的影响、治疗认知、对医患关系的看法。本亚组研究主要对问卷中的症状、ITP对生活质量和情绪的影响、治疗认知这三部分的调研结果进行描述性分析。

调研中的部分维度采用Likert-7分法进行评价，患者根据自身情况对量表中陈述句认同程度进行评分，评分范围为1～7分，分数越高程度越高，1分代表“完全不”，4分代表“一般”，7分代表“程度非常大”。评价健康状态时，分数越高越好，7分代表“非常好”。评价症状严重程度时，分数越高越严重，7分代表“非常严重”，采用严重程度为5～7分的患者所占比例作为某一症状严重程度的指标。评价影响程度时，分数越高影响越大，7分代表“影响非常大”。评价满意程度时，分数越高越满意，7分代表“非常满意”。评价对观点的认可程度，分数越高越认可，7分代表“非常同意”。

## 结果

一、一般资料

共286例患者参与调研，平均年龄为36岁，其中170例（59.4％）为女性，17例（5.9％）接受过脾切除术。65.0％（186/286）的患者报告近期健康状况较好，15.0％（43/286）的患者报告近期健康状况较差（表1）。102名医生参与了调研，其中73名（71.6％）执业超过15年。在参与调研的前12个月内，每名医生平均诊治ITP患者51例。

**表1:** 参与I-WISh国际调研中国原发免疫性血小板减少症（ITP）患者和医生的一般资料

特征	结果
患者（286例）	
女性［例（％）］	170（59.4）
平均年龄（岁）	36
既往脾切除术［例（％）］	
是	17（5.9）
否	249（87.1）
未知	20（7.0）
近期健康状态Likert-7评分［例（％）］	
1～3分	43（15.0）
4分	57（19.9）
5～7分	186（65.0）
医生（102名）	
专业科室［例（％）］	
血液科	93（91.2）
血液肿瘤科	9（8.8）
就职地点［例（％）］	
专科肿瘤中心	2（2.0）
大学附属医院/教学医院	83（81.4）
地区/社区医院	15（14.7）
其他	2（2.0）

二、症状

就频率和严重程度而言，患者就诊时最主要的症状是瘀斑，其次是瘀点和月经过多。治疗后，患者瘀斑、出血等躯体症状得到缓解，而对血小板计数不稳定的焦虑和疲劳则成为患者的主要困扰（图1）。医生认为接诊ITP患者时最常见的症状为瘀斑（84.3％，86/102）、瘀点（83.3％，85/102）、牙龈出血（74.5％，76/102）和月经过多（61.8％，63/102）。与患者相比，医生对于焦虑（12.7％，13/102）和疲劳（29.4％，30/102）的报告不足。

**图1:**
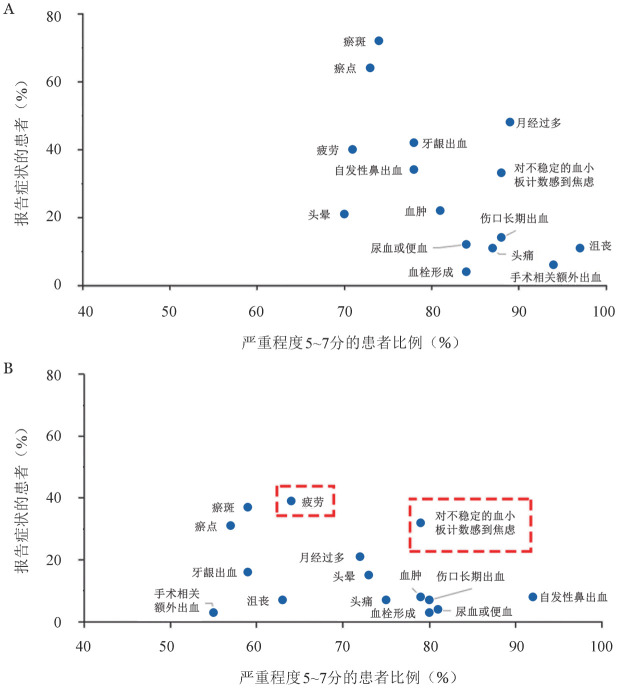
原发免疫性血小板减少症（ITP）诊断时（A）和治疗后（B）的常见症状

三、ITP对生活质量和情绪的影响

97.2％（278/286）的患者认为，ITP对其体能水平有一定影响，其中41.3％（118/286）的患者认为其影响的时间超过一半。90％以上的患者认为ITP对其工作或学习、日常事务、运动能力和性生活产生负面影响，其中40.9％（117/286）的患者认为其运动能力受疾病影响的时间超过一半，35.8％（86/240）的患者认为半数以上时间的工作和学习受到疾病影响（图2）。采用Likert-7分法让医生评价ITP对患者生活质量的影响，6.9％（7/102）的医生认为影响较小（1～3分），9.8％（10/102）的医生认为影响一般（4分），83.3％（85/102）的医生认为影响较大（5～7分）。采用Likert-7分法对患者的情绪状况进行评估，60.8％（174/286）的患者认为ITP对情绪整体影响较大（5～7分），其中对血小板计数的波动（74.8％，241/286）、疾病本身（72.0％，206/286）及病情恶化（68.9％，197/286）的担忧占主要方面。医生认为患者对疾病的恐惧（78.4％，80/102）、对血小板计数波动的焦虑（75.9％，217/286）及对患有ITP的沮丧（71.6％，73/102）是影响情绪的主要因素。因此，医生和患者在ITP对情绪影响的方面和程度上给出了类似评价（图3）。

**图2:**
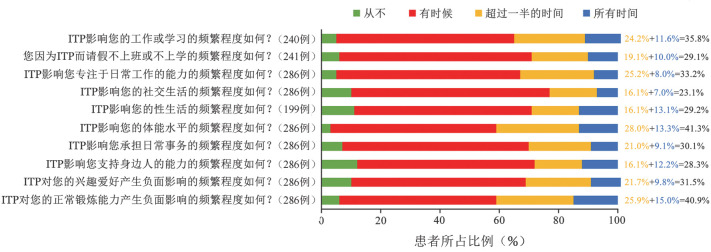
患者报告原发免疫性血小板减少症（ITP）对生活质量的影响

**图3:**
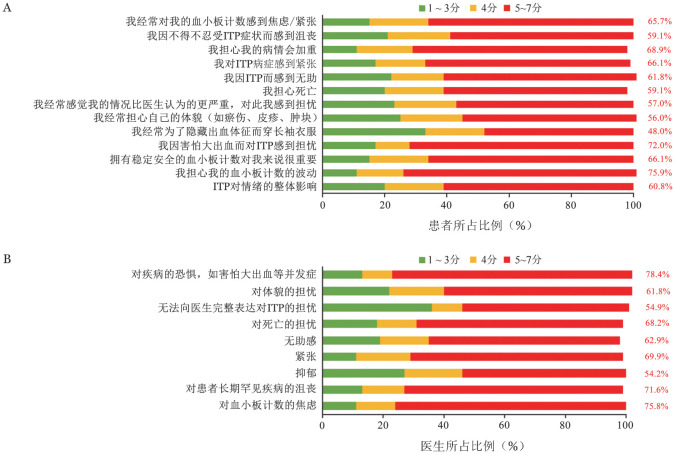
患者（A）和医生（B）认为原发免疫性血小板减少症（ITP）对情绪的影响

对于ITP相关情绪问题的处理，仅31.8％（91/286）的患者认为已经获得了专业治疗，67.8％（194/286）的患者缺少情绪问题的专业治疗，其中72.0％（206/286）的患者希望得到更多专业治疗。20.6％（21/102）的医生在临床工作中会应用自评问卷评估患者的生活质量，且认为有必要开发针对ITP患者生活质量的问卷，64.7％（66/102）的医生表示在今后ITP患者诊治过程中愿意应用该类问卷，14.7％（15/102）的医生仍不考虑应用该类问卷。已经应用或今后愿意应用生活质量问卷的87名医生中，36.8％（32/87）的医生将考虑在每次随访时应用，58.6％（51/87）的医生考虑每6个月使用1次，4.6％（4/87）的医生每年使用1次。86.3％（88/102）的医生认为智能手机应用能够帮助追踪患者的生活状况。

四、治疗认知

ITP患者从症状出现到就诊的平均时间为5.7个月。49.0％（140/286）的患者报告，医生曾采用观察随诊的治疗策略，观察的平均时长是0.4个月。

患者和医生均认为减少自发出血［59.1％（169/286）的患者及75.5％（77/102）的医生］、维持安全稳定的血小板水平［51.0％（146/286）的患者及47.1％（48/102）的医生］、改善生活质量［47.9％（137/286）的患者及58.8％（60/102）的医生］是ITP最重要的三个治疗目标（详见表2）。

**表2:** 参与I-WISh国际调研中国原发免疫性血小板减少症（ITP）患者和医生的治疗目标［例（％）］

治疗目标	患者（286例）	医生（102名）
减少自发出血	169（59.1）	77（75.5）
维持安全稳定的血小板水平	146（51.0）	48（47.1）
改善生活质量	137（47.9）	60（58.8）
预防疾病恶化，延缓进展	117（40.9）	41（40.2）
减轻症状	94（32.8）	27（26.5）
提高体能	83（29.0）	/
减少经期出血	46（16.1）	5（4.9）
改善情绪	37（12.9）	/
减少全身无力	20（6.9）	11（10.8）
预防血栓形成	12（4.2）	7（6.9）
减轻疲劳	/	18（17.6）
简化治疗方式	/	12（11.8）

注：/：不适用

患者和医生的治疗选择倾向基本一致，均优先考虑能够降低出血风险、持续保持疗效或治愈、不良反应最小的治疗方案（表3）。

**表3:** 医生（102名）和患者（286例）对影响原发免疫性血小板减少症（ITP）治疗决策相关因素的重要性评分

因素重要性平均分（1～100分）	患者平均评分	医生平均评分
持续应答或治愈	89	79
降低出血风险	88	87
不良反应最小化	87	76
较慢恢复，但可能是长期的	74	69
快速恢复，但可能只是暂时性的	69	69
预防免疫抑制	65	61

针对ITP患者不同的血小板计数水平，医生采取的治疗方案如下：对于血小板计数>50×10^9^/L的患者，52.0％（53/102）的医生将观察随诊作为主要治疗方式之一；对于血小板计数<10×10^9^/L的患者，分别有70.6％（72/102）、62.7％（64/102）、56.9％（58/102）的医生将静脉注射免疫球蛋白（IVIg）、糖皮质激素、血小板输注作为主要治疗方式，反映了我国医生对血小板极低患者可能发生危及生命出血的高度重视。

医生认为糖皮质激素最有可能使患者达到持续有效［54.9％（56/102）］，脾切除术（42.2％，43/102）、利妥昔单抗（40.2％，41/102）、血小板生成素受体激动剂（TPO-RA）（40.2％，41/102）使患者获得持续有效的比例相近。若以持续有效为目标，55.9％（57/102）的医生会选择糖皮质激素，15.7％（16/102）的医生会选择TPO-RA（表4）。

**表4:** 参与I-WISh国际调研的102名中国医生对原发免疫性血小板减少症（ITP）的治疗认知［％（例）］

治疗	认为最可能使患者达到持续有效治疗	更倾向于选择以持续有效为治疗目标
糖皮质激素	54.9（56）	55.9（57）
脾切除术	42.2（43）	8.8（9）
抗CD20抗体	40.2（41）	14.7（15）
TPO-RA	40.2（41）	15.7（16）
静脉注射免疫球蛋白	36.3（37）	3.9（4）
其他免疫抑制剂	35.3（36）	0.9（1）
其他治疗	16.6（17）	0（0）

注：TPO-RA：血小板生成素受体激动剂

286例患者中，17例（5.9％）接受了脾切除治疗，其中59.1％（169/286）的患者报告不同程度的不良后果（腹部疼痛、呼吸道感染增多等），29.0％（83/286）的患者需定期接种疫苗。采用Likert-7分法对脾切除术后患者的感受进行评价，45.1％（129/286）的患者对脾切除治疗效果满意（5～7分），59.1％（169/286）的患者因无效而后悔接受脾切除术（5～7分），33.9％（97/286）的患者认为他们并未对脾切除术的长期并发症充分知情（1～4分）。采用Likert-7分法让患者提供治疗反馈，整体而言，患者对TPO-RA的反馈更积极。大部分患者认为TPO-RA能有效预防出血事件（5～7分）（88.1％，252/286）、减轻症状（5～7分）（75.2％，215/286）和提高体能（5～7分）（72.0％，206/286）并且会推荐给其他ITP患者（5～7分）（76.9％，220/286）。而对糖皮质激素疗效满意的患者比例（5～7分）（58.0％，166/286）低于TPO-RA（5～7分）（83.9％，240/286）、利妥昔单抗（5～7分）（69.9％，200/286）和IVIg（5～7分）（73.1％，209/286），认为糖皮质激素可以预防出血事件（5～7分）（61.9％，177/286）、减轻症状（5～7分）（60.8％，174/286）和提高体能（5～7分）（46.9％，134/286）的患者比例也低于TPO-RA。

## 讨论

ITP是一种临床表现多样的异质性疾病，多种症状会对患者的情绪和生活质量产生严重且长期的影响。I-WISh调研[5]与我国亚组分析均表明疲劳和对血小板计数不稳定的焦虑是困扰患者的主要因素。瘀伤在治疗后虽然得到明显缓解，但仍然是常见症状之一，不明原因的瘀伤容易使患者在社交场合感到不适[6]。对于慢性病患者而言，疲劳是最常见和令人痛苦的症状之一[1],[7]，常导致工作能力下降[8]–[9]。由于目前ITP临床效果评估多使用血小板计数作为主要指标，且多数患者对低血小板计数相关后果有清晰认知，因此，即便不存在严重出血症状，低血小板计数本身就可引起患者的持续焦虑[2]，而焦虑情绪也会进一步加重患者的疲劳感[7]，最终对患者的生活质量造成严重影响。值得注意的是，在患者随访时，医生往往更多关注紫癜和月经过多等躯体症状，对于患者的疲劳感和焦虑症状关注相对不足。2019版ITP诊治国际共识报告更新[10]指出，医生在接诊ITP患者时应关注其疲劳症状和心理健康。

全球I-WISh调研[5]中，49％的患者认为ITP对其情绪有较大影响（≥5分），63％的患者担心病情恶化及血小板计数的波动；而我国亚组显示，60.8％的患者认为ITP对情绪影响较大，分别有68.8％和74.8％的中国患者担忧其病情恶化和血小板计数波动，提示我国有更高比例的患者其情绪状况受ITP影响，也体现患者对稳定血小板计数的需求。ITP患者和医生均认为该疾病可引发焦虑、抑郁和恐惧等负面情绪，虽然大部分医生知晓ITP对患者情绪状况的影响，但仅31.8％的患者得到了专业治疗，提示医生在诊疗过程中应加强对患者的心理疏导，通过患者教育提升其对疾病的认知。与心理科的合作也可能为患者提供更多帮助。

慢性ITP患者与疾病长期共存，因此对疾病的医疗干预不仅需要缓解躯体症状，还应改善患者的生活质量[11]。应用Likert-7分法评价ITP对生活质量的影响时发现，ITP在日常生活不同方面均给患者造成一定程度的负担。超过90.0％的患者其工作或学习、日常事务、体能、运动能力和性生活等受到影响，其中40.9％的患者认为其日常体能和运动能力受ITP影响的时间超过一半。在I-WISh全球调研[5]中，体能和运动能力受影响的时间超过一半的患者则分别占44％和36％。2019版ITP诊治国际共识报告指出，ITP患者的健康相关生活质量受到严重影响，需进一步提高医生对ITP患者生活质量的关注度[10]。基于患者报告的临床结局对ITP患者临床评价有重大意义[10]。因此，联系患者定期反馈治疗后的生活质量改善情况，针对性地调整治疗措施可使患者受益。中国亚组数据中发现，仅20.6％的医生正在使用问卷评估患者的生活质量，64.7％的医生愿意在今后使用相关问卷。结合我国国情，一方面原因是由于诊疗时长的限制，医生无法为每位患者提供较长的时间完成问卷，另一方面原因可能是目前缺乏简单有效且针对ITP患者生活质量的随访工具。考虑到86.3％的医生认为智能手机应用能够帮助跟踪患者预后，因此有必要开发相应的应用软件，在高效随访的同时增进医患沟通。

在选择治疗方式时，医生与患者均优先考虑能持续降低出血风险的方法，并尽量避免不良反应和免疫抑制的发生。在全球I-WISh调研[12]中，当患者血小板计数>30×10^9^/L时，40％以上的医生将观察随诊作为主要治疗方式之一，这一界值在中国亚组数据中是50×10^9^/L。对于血小板计数<10×10^9^/L的患者，半数以上中国医生会对患者进行血小板输注治疗，提示我国医生对血小板计数的管理预期更高。在治疗方式的疗效认知上，中国医生认为相对于TPO-RA（40.2％）、脾切除术（42.2％）和利妥昔单抗（40.2％），接受糖皮质激素（54.9％）治疗的患者达到持续有效的概率更高,这与既往文献报道的大多数中国医生会把糖皮质激素作为主要治疗的结果相一致[13]。随着有确切循证证据的药物治疗种类增加，以及对脾切除术的长期预后及远期并发症的进一步认识[14]–[15]，ITP患者脾切除术的应用逐渐减少。中国患者脾切除比例（5.9％）较全球比例（20％）[12]更低，其中仅45.1％的患者对脾切除术的总体疗效满意，这表明我国患者对侵入性、不可逆且可能伴有术后并发症的脾切除术接受度更低。TPO-RA类药物通过激活血小板生成素受体及其下游通路，增加血小板的生成[16]。全球I-WISh数据[12]表明，以ITP持续有效为目标，选择TPO-RA治疗的医生更多（32％），而我国医生更倾向于选择糖皮质激素（55.9％）作为达到持续有效的治疗手段。83.9％的国内患者对TPO-RA治疗的总体疗效表示满意，因此我国医生可提高TPO-RA的使用率。2019版美国血液学会（ASH）ITP指南[17]指出，二线治疗方案应该根据疾病持续时间、需要住院或抢救治疗的出血发作频率、并发症、患者年龄、药物依从性、医疗和社会支持网络、患者价值观和偏好、费用和可用性等因素进行个体化选择，同时应重视患者教育和鼓励医患共同决策。而在对不同治疗方案的不良反应的反馈上，医患双方存在的差异也提示了医患共同决策的重要性。

总之，ITP是一种慢性疾病，具有持续的出血风险，合理的治疗方法有助于改善患者的预后并提高患者生活质量。我国ITP患者的疲劳和焦虑症状突出，生活质量受到疾病负面影响的程度高于全球数据，因此在临床诊治中应给予更多关注。此外，虽然我国医患双方对ITP的诊治目标相同，但医生倾向使用的治疗方式和患者反馈的最佳治疗方式有所不同，这提示在治疗方式的选择上医患双方需要相互沟通，进一步体现了医患共同决策的重要性。
